# Clinical, pathological, and genotypic analysis of infectious bronchitis virus in broiler chickens in the Abu Dhabi Emirate, United Arab Emirates

**DOI:** 10.3389/fvets.2024.1474181

**Published:** 2025-01-27

**Authors:** Hassan Zackaria Ali Ishag, Abdelnasir Mohammed Adam Terab, Ebrahim Mohamad Abdalsalam Osman, El Tigani Ahmed El Tigani-Asil, Mohammed Saleh Albreiki, Oum Keltoum Bensalah, Asma Abdi Mohamed Shah, Abdelmalik Ibrahim Khalafalla

**Affiliations:** ^1^Biosecurity Affairs Division, Development and Innovation Sector, Abu Dhabi Agriculture and Food Safety Authority, Abu Dhabi, United Arab Emirates; ^2^Animals Extension and Health Services Division, Abu Dhabi Agriculture and Food Safety Authority (ADAFSA), Abu Dhabi, United Arab Emirates; ^3^Applied Research and Capability Building Division, Abu Dhabi Agriculture and Food Safety Authority, Abu Dhabi, United Arab Emirates

**Keywords:** infectious bronchitis virus, clinical, pathology, lineage, United Arab Emirates

## Abstract

**Background:**

Infectious Bronchitis (IB), caused by the infectious bronchitis virus (IBV), is a significant contagious respiratory disease in the poultry industry. The emergence of new variants represents a global challenge for the diagnosis and control of the disease. Despite vaccination efforts in poultry farms in the Abu Dhabi Emirate, United Arab Emirates (UAE), outbreaks continue to occur, raising concerns about the efficacy of vaccination protocols and the potential emergence of new viral strains. This study aims to provide information on clinical, pathological, and genotypes of IBV detected within the Abu Dhabi Emirate, during 2022–2023.

**Methods:**

Epidemiological data were collected from twelve suspected IB outbreaks across seven broiler farms located in the Abu Dhabi Emirate. The cases were investigated through clinical and pathological examinations and Forty-six samples, including lung, spleen, kidney tissues, and oro-cloacal swabs, were collected for further analysis. The virus was detected by RT-qPCR assay, genotyping was determined by phylogenetic analysis of the Spike (S)-1 gene, and differentiation between field and vaccine strains was determined by comparing their sequences.

**Results:**

The age of the affected flocks varies from 2 to 5 weeks. The highest morbidity, mortality and case fatality rates were 36, 33, and 95%, respectively. Necropsy examination revealed characteristic respiratory and renal pathological lesions. Phylogenetic analysis revealed a co-circulation of three lineages of IBV genotype GI-13 or 4/91 serotype (81.8%), GI-1 or Massachusetts serotype (9.1%) and GI-23 or Middle East serotype (9.1%). Approximately 90.9% of the strains classified within GI-1 and G1-13 lineages are 99 to 100% identical to 4/91 and Mass serotypes, respectively, and are considered as vaccine strains. Two strains (9.1%) classified within GI-23 lineage have a < 99% identity to the 4/91 and Mass serotypes vaccine strains and are considered as filed strains.

**Conclusion:**

Co-circulation of three IBV lineages (GI-13, GI-1, and GI-23) in the Abu Dhabi broiler flocks showing IB symptoms were detected. This complex scenario of different IBV lineages circulation may account for the persistent outbreaks despite vaccination efforts. The results of the study are crucial for optimum IB vaccination and monitoring strategies or designing new vaccines based on local IBV field strains.

## 1 Introduction

Avian infectious bronchitis (IB) represents a persistent challenge in the poultry industry (1, 2), as it induces significant economic losses (3). The disease, caused by the infectious bronchitis virus (IBV) (4, 5), which is a member of the Gammacoronavirus genus within the family of Coronaviridae (6). As a highly contagious pathogen, the virus affects multiple physiological systems in chickens, including respiratory tract, kidneys, and the reproductive organs, affecting all age groups of chickens (7). Clinically, the infected chickens manifests diverse symptoms ranging from respiratory signs such as coughing, sneezing, nasal discharge to reproductive signs like reduced egg production, eggshell deformities, and the occurrence of false layers (8, 9). In young birds, the mortality rate can reach up to 20–30%, while the morbidity often approcahes100% (10). The disease severity is increased by secondary bacterial and viral infections including *Escherichia coli* (*E. coli*), infectious bursal disease virus (IBDV) and Marek’s disease virus (10), further complicating the IB management.

The IBV transmission occurs through direct contact with infected birds or indirectly through contaminated feed, water, aerosol droplets and other fomites (11, 12). Pathological changes frequently reported in the respiratory and urinary systems include trachea congestion and mucus depositions, while kidneys showed enlargement and discoloration with the presence of whitish materials (13–15). These clinical and pathological finding of the disease, however are not definitive, warranting further epidemiological investigations and laboratory confirmation of the disease (16, 17).

Vaccination remains the most effective approach for IB prevention, relying primarily on live-attenuated and killed vaccines formulations (18). Despite their widespread use, outbreaks of IB persist in vaccinated flocks, often attributed to antigenic variations between vaccine strains and emerging field strains. In some instances, live vaccine strains have been implicated in numerous IB outbreaks (19, 20), highlighting the complexity of the disease management.

A critical component of IBV control is understanding its genetic diversity. This is typically achieved through sequencing and phylogenetic analysis of the spike 1 (S1) gene, which is part of spike glycoprotein (S) that is cleaved into two proteins (S1 and S2) during the post-translational modification (21, 22). The S1 protein, a determinant of the virus serotype and immunogenicity, frequently undergoes genetic modifications, including amino acid substitutions, insertions, and deletions (22). These changes can lead to the emergence of novel serotypes, vaccination failure and necessitating the development of new homologous vaccines. Even minor variations in the S1 subunit, such as substitutions of 10–15 amino acids (2–3%), can generate serotypes distinct from those targeted by existing immunization programs (23, 24).

Based on the complete sequences of the S1 gene, the IBV strains were grouped into 9 major genotypes (GI- GIX) (24, 25). The GI genotype contained 1 to 31 lineages (24–26), while each of the other genotypes has only one lineage. Lineages such as GI-1 (Massachusetts or Mass type), GI-13 [4/91 (793B or CR88)-like], GI-19 (LX4 or QX), GI-16 (Q1), GI-21 (Italy02) are globally distributed (27), and are the most commonly used as vaccine strains. In contrast, the GI-23 lineage (Is-Variant2, also known as Middle East type) and its variants, is predominantly found in the Middle East (24), and currently endemic across Europe and Asia, with no homologous vaccine available in use (24, 28–32).

In Middle Eastern countries, there have been numerous sporadic reports of IB from different countries, including Jordan (33), Egypt (34), Iran (35), Libya (36) and Oman (37) with the most prevalent serotype being 4/91 or 793B (43.66%), representing the GI-13 lineage. Co-circulation of different IBV lineages was observed in some countries such as the co-existence of GI-23, GI-1, GI-12, GI-13, GI-19 lineages were reported in Iran (38, 39), whereas the GI-13 or 4/91 IBV (31%), GI-16 or CK/CH/LDL/97I IBV (28.6%), GI-1 or Mass IBV (19%), and GI-23 or Middle East IBV (21.4%) were reported in Saudi Arabia (40). In the UAE (2010–2014), the 793/B (GI-13) strain represents the common genetic lineage that infects chicken flocks, while the Mass (GI-1) and D-274 (GI-12) strains were the least widespread genotypes detected (28, 41).

In the Abu Dhabi Emirate, IBV outbreaks have been reported despite vaccination practices using the Mass serotype alone or combined attenuated vaccines (Ib4/91 + Ma5 or Ma5 clone 30). Between 2022 and 2023, multiple outbreaks occurred in broiler farms, including vaccinated ones, leading to significant economic losses. Despite these challenges, the genotyping of the circulating IBV strains in Abu Dhabi farms and its relationship with global strains has not been evaluated, which represents a gap in vaccination strategies and disease control requirements. Here, we undertook epidemiological, clinical, pathological, and molecular investigations followed by sequencing and phylogeny of the S1 gene to diagnose and genotype the IBV in seven commercial broiler farms that presented clinical signs of IB. We further investigated whether the UAE-IBV strains belonged to field or vaccine strains. To our knowledge, this is the first comprehensive study to describe and genotype IBV in the UAE.

## 2 Materials and methods

### 2.1 Epidemiological data, necropsy, and sampling

**Figure 1:**
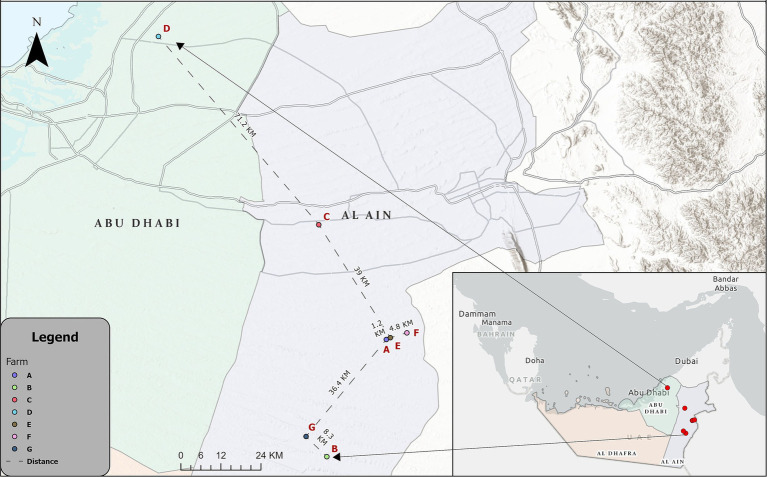
Map showing the location of the IBV-infected farms in the Al Ain region [Farms **(A–F)**] and Abu Dhabi region [Farm **(D)**].

Twelve IB outbreaks that occurred in broiler farms in the Abu Dhabi Emirate between August 2022 (*n* = 6) and May 2023 (*n* = 6) were reported by Abu Dhabi Agriculture and Food Safety Authority (ADAFSA) veterinarians, along with case histories, epidemiological information, and clinical symptoms. These outbreaks, originating from seven different farms, six farms (labeled A, B, C, E, F, and G) are located in the Al Ain region, while one farm (farm D) is located in the Abu Dhabi region (Figure 1; Table 1). Some farms experienced repeated infections, with Farm C encountering two occurrences and Farm A experiencing five episodes. Suspected IBV cases (seventy-nine broiler chickens of different ages and disease stages, as well as recently dead chickens) collected from different farms were submitted to ADAFSA Veterinary Laboratories for necropsy examination and subsequent laboratory analysis (Table 1).

**Table 1:** Details of IBV outbreaks and samples collected in the current study.

Farm	Year	Outbreak	Postmortem and sampling date	Total birds necropsied	Total birds sampled	Sample type
A*	2022	1	31.08.2022	3	2	Lung = 2
B		2	12.09.2022	4	2	Lung = 2 Kidney = 2
C#		3	17.10.2022	9	3	Lung = 3
A*		4	09.12.2022	7	6	Spleen = 4 Oro-cloacal swab = 2
		5	20.12.2022	8	3	Kidney = 3
D		6	21.12.2022	6	2	Lung = 2 Kidney = 2
E	2023	7	08.02.2023	4	2	Lung = 2
F		8	28.02.2023	13	3	Lung = 3
A*		9	27.02.2023	8	3	Lung = 3
G		10	08.03.2023	8	3	Lung = 3 Kidney = 3
A*		11	13.03.2023	6	2	Lung = 2 Kidney = 2
C#		12	05.05.2023	3	3	Lung = 3 Oro-cloacal swab = 3
Total				79	34	46

A*A* & C# denotes for recurrent infections in the specified farms.

Gross lesions were documented, and tissue samples from different chickens, including lung, kidney, trachea, and spleen, were collected and fixed in 10% neutral formalin for histopathological analysis. Following necropsy examinations, 46 tissue and swab samples, including lung (*n* = 25), spleen (*n* = 4), kidney (*n* = 12), and oro-cloacal swabs (*n* = 5) were collected from 34/79 (43%) necropsied broiler chickens. IBV-suspected broiler chickens (Table 1) were refrigerated and shipped to the ADAFSA veterinary laboratory in Abu Dhabi for RT-qPCR confirmatory testing. All tests performed in this study were in the context of routine diagnosis and clinical activities, with no experimental treatments or additional assays applied during the study period. The approval of the use of samples and animals for research was obtained from the owner before their inclusion in the study.

### 2.2 Histopathological examination

Tissue samples, including lung, kidney, trachea and spleen, were fixed in 10% neutral formalin for 24–48 h at room temperature and processed for histopathological examination according to the standard methods (42). Formalin-fixed tissue samples were processed in an automatic tissue processor (ATP1-220, Triangle Biomedical Sciences, Durham, USA), embedded in paraffin wax, and cut into 5 μm thick sections. Histologic sections were stained with Hematoxylin and Eosin (H & E) (Thermo Fisher Scientific, Runcorn, Cheshire, UK), then examined under a microscope, described and images were captured using the VisionTek digital microscopy system (DM01, Sakura Finetek, Torrance, USA).

### 2.3 Real-time quantitative PCR (RT-qPCR) based detection of IBV

Total RNA extraction from tissues or swabs was carried out using the EZ1 Virus Mini Kit V2.0 (43) (Qiagen, Hilden, Germany) on the Advanced EZ1 instrument (Qiagen in Hilden, Germany) following the manufacturer’s instructions. The presence of IBV RNA was detected by RT-qPCR targeting the 5’ UTR gene of IBV as previously described (16). The forward primer (IBV5 GU391: 5′-GCTTTTGAGCCTAGCGTT-3′), the reverse primer (IBV5 GL533: 5′- GCCATGTTGTCACTGTCTATTG-3′), and TaqMan probe (IBV5 G probe: FAM-5′- CACCACCAGAACCTGTCACCTC-3′-BHQ1) were utilized for this purpose. The Real-time ready RNA Virus Master kit (Roche) was used for preparing the reaction mix, consisting of 7.9 μL of water, 0.1 μL 50 × enzyme mix, 4.0 μL of 5 × reaction buffer and 1 μL of each primer solution (10 pmol/μL). The RNA template was added at 5 μL to complete the volume of 20 μL. The thermal cycling profile initial steps of 50°C for 30 min, and 95°C for 5 min, followed by 40 cycles of amplification (94°C for 1 s and 60°C for 1 min). RT-qPCR analysis was conducted using the BioRad CFX 96 Touch Real-Time PCR Instrument (BioRad).

### 2.4 Amplification of S1 gene, sequencing, and phylogenetic analysis

#### 2.4.1 Amplification of S1 gene

The 464 bp hyper-variable region of the S1 gene was PCR amplified using the SuperScript ™ III Platinum™ One-Step RT-PCR Kit (Thermo Fisher, Waltham, MA, USA), following a previously described method (44) with minor modifications. In brief, we combined 5 μL of extracted RNA with a standard mix containing 1× Reaction mix, XCE1 + (CACTGGTAATTTTTCAGATGG) and XCE2 (CTCTATAAACACCCTTACA) primers at a concentration of 10 pmol/μL, and 1 μL of SuperScript™ III RT/Platinum™ Taq Mix (Thermo Fisher, Waltham, MA, USA). Then, we added molecular-biology-grade water to make a final volume of 25 μL and proceeded with PCR thermal cycling under the following conditions: 50°C for 30 min, 95°C for 2 min, followed by 45 cycles of 95°C for 15 s, 50°C for 20 s, and 68°C for 40 s. A final extension step was performed at 68°C for 5 min. The amplicon was visualized in a 1.8% agarose gel.

#### 2.4.2 Sanger sequencing and BLAST analysis

The amplicon was Sanger-sequenced (bi-directional) at ADAFSA laboratory using the same primers as for PCR. First, we used the ExoSAP-IT™ PCR Product Cleanup Reagent (Thermo Fisher Scientific) to purify the PCR products of the S1 gene, and BigDye™ Terminator v3.1 Cycle Sequencing kit (Applied Biosystems) was utilized to perform the Sanger sequencing as previously described (44). The reaction mixture totaling 20 μL comprised 9 μL of water, 3.5 μL of 5× Sequencing Buffer, 1 μL of the BigDye™ Terminator v3.1, 1 μL of primers (3.2 pmol/μL), and 5.5 μL of DNA. Next, we purified the reaction mixture using the BigDye™ XTerminator™ Purification kit (Applied Biosystems) according to the manufacturer’s instructions. Sequencing was carried out on a SeqStudio Genetic Analyzer (Applied Biosystems) using the ‘MediumSeq BDX’ run module. The obtained sequences were trimmed and assembled using CLC Genomic Workbench v.20 (Qiagen, Aarhus, Denmark). Then we subjected the consensus sequences of the S1 gene to BLAST analysis using the BLAST Tool at NCBI GenBank (Basic Local Alignment Search Tools)^1^ to confirm the sequence identity.

#### 2.4.3 Sequence alignment and phylogenetic analysis

The S1 gene of the IBV-UAE strains was aligned using the ClustalW program in MEGA 11 (45). This alignment included sequences from UAE-IBV and corresponding sequences of reference strains from all the IBV lineages, totaling 234 sequences. These sequences were sourced from the NCBI nucleotide sequence database or obtained from the publication by Valastro et al. (24). Commonly used vaccine strains were also integrated into the analysis. A phylogenetic tree was constructed using the Maximum Likelihood technique and the Kimura 2-parameter model (46) within the MEGA 11 program (47). The pairwise nucleotide sequence similarity within the UAE - IBV S1 gene was then determined.

#### 2.4.4 Differentiation of filed versus vaccine strains of UAE – IBV

At present, there is no definitive approach to distinguish between vaccine and field viruses of the same genotype, except for a method that involve comparing the genetic sequences of the detected viruses with those of established vaccine strains (9, 17). In this method, strains exhibiting 99–100% similarity with commercial vaccine strains were categorized as vaccine strains, while those showing less than 99% similarity were considered field strains. Accordingly, we compared the sequences of UAE - IBV strains identified in this study with those of standard vaccine types, including Mass-type and 4/91 live-attenuated vaccine strains.

## 3 Ethical approval

This research was approved by the research ethics committee Abu Dhabi Agriculture and Food Safety Authority (ADAFSA) (approval number: ADAFSA-EA-09-2023), and the study was conducted following the guidelines stated for animal use. A written consent (which was included in the sample request form approved by the ADAFSA research ethics committee) was obtained for the use of samples and animals from the owner before inclusion in the study.

## 4 Results

### 4.1 Outbreaks investigations

Twelve outbreaks of IBV occurred in seven broiler farms located in Al Ain and Abu Dhabi regions of the Abu Dhabi Emirate (Table 1) from August 2022 to May 2023. Some farms experienced recurring outbreaks. Notably, farm A had five outbreaks within a two-year period (three outbreaks in 2022 and two in 2023 with intervals of approximately one to two weeks). Farm C also experienced two outbreaks, one in October 2022 and another in May 2023. The broiler population per farm ranged from 14,500 to 37,000 chickens (Table 2). Affected flocks showed various clinical signs including diarrhea, respiratory signs, and high mortality. The age of the infected flocks varied from 2 to 5 weeks. In 2022, the highest morbidity (36%) and mortality (33%) rates were observed in farm C, whereas the highest case fatality (95%) rate was observed in farm D. In contrast in 2023, Farm C was severely affected where the highest (19, 18, 93%) morbidity, mortality and case fatality rates were observed, respectively. All affected farms were vaccinated based on the Ma5 serotype alone or combined Ib4/91 + Ma5 clone 30 live attenuated vaccines. The duration between the vaccination and disease onset remains unknown. Importantly, there was no recent history of introducing new, unvaccinated flocks across all affected farms prior to the outbreaks (Table 2). The information for farms B and G regarding this matter is unavailable.

**Table 2:** Epidemiological information of IBV outbreaks reported in the Abu Dhabi Emirate in 2022–2023.

Farm	Year	Outbreak	Lab registration date	No. of samples sequenced	GenBank Acc. No	Total population	Total infected	Age (day)	Death	Morbidity rate	Mortality rate	Case fatality rate	Vaccination status of infected farms	Vaccine type	Introduction of new unvaccinated flocks
A*	2022	1	01.09.2022	1	OR161340	30,000	3,000	14	2000	10%	7%	67%	Vaccinated	ma5	No
B		2	14.09.2022	NA	NA	34,000	4,000	20	2,500	12%	7%	63%	Vaccinated	ma5	--
C#		3	20.10.2022	2	OR161341 OR161342	33,000	12,000	17	11,000	36%	33%	92%	Vaccinated	Ib4/91 + ma5 clone 30	No
A*		4	13.12.2022	6	OR161343 to OR161348	33,000	4,000	34	2,500	12%	8%	63%	Vaccinated	ma5	No
			13.12.2022												
		5	22.12.2022	1	OR161349										
D		6	22.12.2022	1	OR161350	14,500	2,300	30	2,184	16%	15%	**95%**	Vaccinated	Ib4/91 + ma5 clone 30	No
E	2023	7	10.02.2023	1	OR161351	30,000	5,000	36	4,000	17%	13%	80%	Vaccinated	Ib4/91 + ma5	No
F		8	01.03.2023	2	OR161352 OR161353	21,000	3,000	17	2000	14%	10%	67%	Vaccinated	Ib4/91 + ma5	No
A*		9	01.03.2023	1	OR161354	30,000	2000	10	1,500	7%	5%	75%	Vaccinated	ma5	No
G		10	08.03.2023	1	OR161355	20,000	3,000	17	2000	15%	10%	67%	Vaccinated	ma5	--
A*		11	14.03.2023	3	OR161356 OR161357 OR161358	30,000	4,000	14	3,000	13%	10%	75%	Vaccinated	ma5	No
C#		12	08.05.2023	3	OR161359 OR161360 OR161361	37,000	7,000	17	6,500	19%	18%	93%	Vaccinated	Ib4/91 + ma5 clone 30	No

--, Information is not available; NA, not assessed.

### 4.2 Pathological analysis

**Figure 2:**
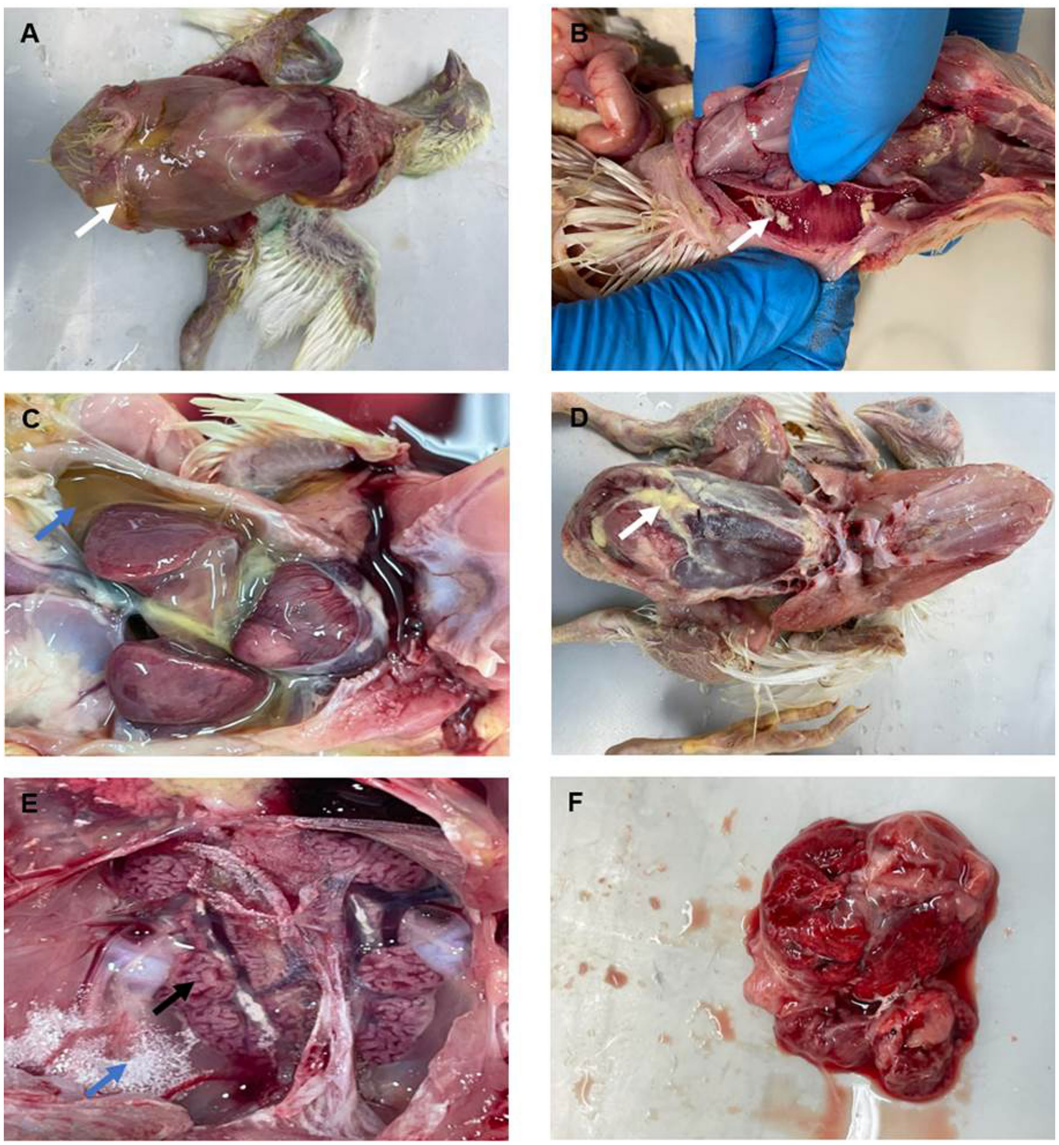
Gross lesions observed in organs of broiler chickens infected with IBV. **(A)** Abdomen exhibiting distension with yellowish exudates (arrow). **(B)** Tracheal mucosa showing congestion, hyperemia, and the presence of yellowish fibrinous materials (arrow). **(C,D)** Coelomic cavity, revealing yellowish fluids (blue arrow) and fibrinous exudates, with adhesions to the surfaces of the liver and heart (white arrow). **(E)** Kidneys appearing swollen and congested, with distended tubules (black arrow) with urates deposits (blue arrow). **(F)** Lungs showing signs of pneumonia with congestion and edema.

Necropsy examination of chickens (*n* = 46) revealed prominent digestive, respiratory and renal gross lesions including distention of the abdomen with yellowish exudates (Figure 2A), congestion, hyperemia, and the presence of a white-yellowish fibrinous diphtheric plug in the trachea (Figure 2B). Yellowish exudates and fibrin clots were also observed in the coelomic cavity, while the surfaces of the liver and heart were covered with pale fibrinous materials (Figures 2C,D). The kidneys appeared swollen with whitish distended tubules and deposits of urates (Figure 2E), while the lungs showed signs of pneumonia with congestion and edema (Figure 2F).

### 4.3 Histopathological findings

**Figure 3:**
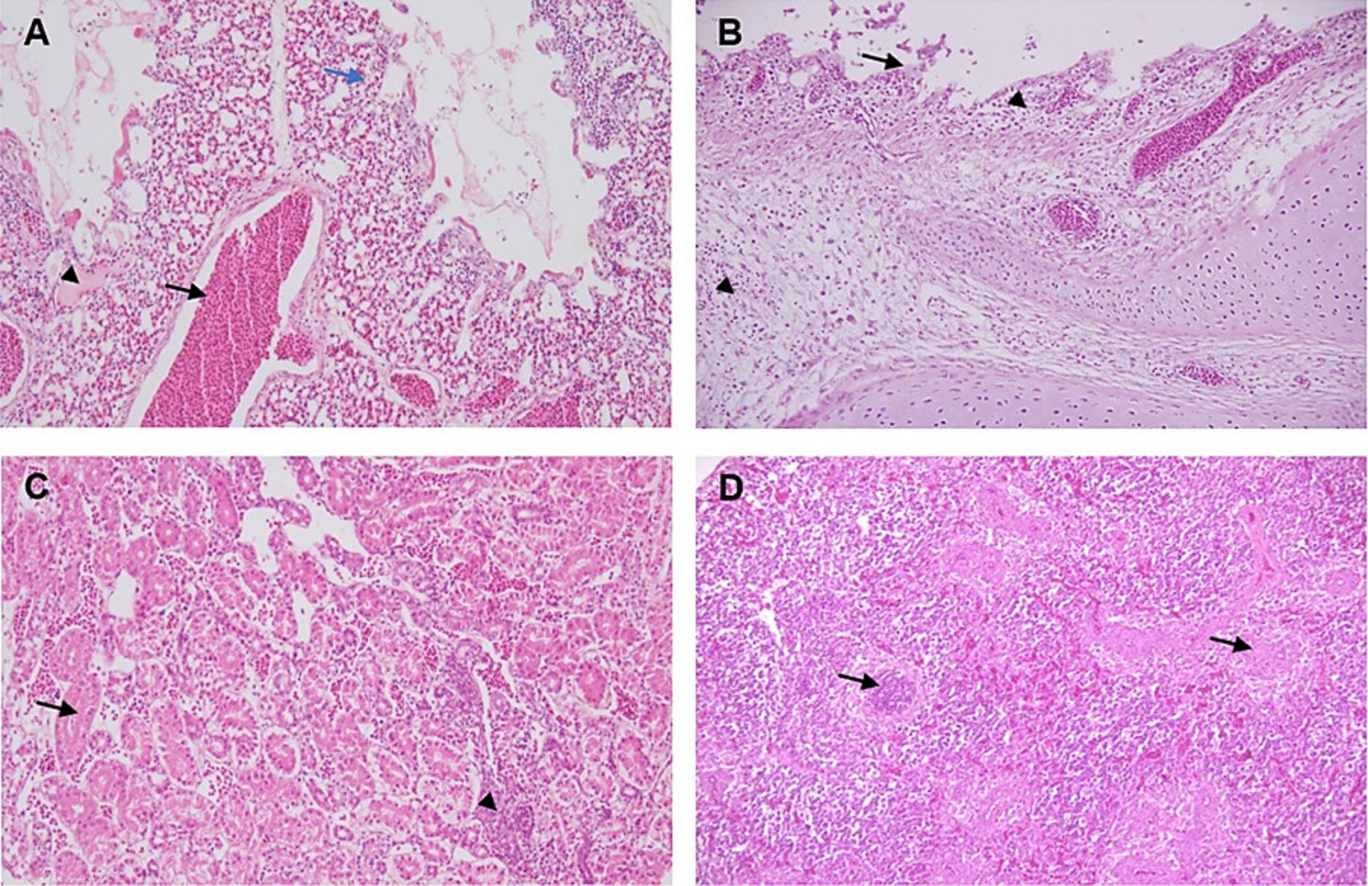
Histopathological lesions in the lung, trachea, kidney, and spleen of IBV-infected broiler chicken. H&E staining, 200×. **(A)** Lung tissue displaying congestion (black arrow), hemorrhage, and edema (arrowhead), accompanied by thickening of the inter-alveolar areas (blue arrow), and a slight infiltration of inflammatory cells. **(B)** The trachea showing degenerative and necrotic changes of the surface epithelium and sloughing (arrow), with mononuclear inflammatory cell infiltrations within mucosa and lamina propria (arrow heads). **(C)** Kidney section showing interstitial lymphocytic infiltration (arrow head), along with degeneration and necrosis of the tubular epithelium (arrow). **(D)** Spleen showing focal necrotic areas with depletion of lymphocytes (arrows).

Microscopic findings of the lungs comprised congestion, hemorrhage, and edema, characterized by thickening of the inter-alveolar spaces and infiltration of inflammatory cells (Figure 3A). The trachea showed degenerative and necrotic changes of the surface epithelium and sloughing, with mononuclear inflammatory cell infiltrations within mucosa and lamina propria (Figure 3B). Kidney sections exhibited focal interstitial lymphocytic infiltration, along with degeneration and necrosis of the tubular epithelium (Figure 3C). The spleen showed focal necrotic areas with depletion of lymphocytes (Figure 3D).

### 4.4 Real-time quantitative PCR

The RT-qPCR analysis revealed the presence of IBV RNA in 84.8% (39/46) of samples collected from different outbreaks. The distribution of positive samples per outbreak is presented in Table 3. The Ct values of the samples ranged between 15 and 34.

**Table 3:** Real-time PCR findings from the analysis of 46 samples across 12 outbreaks.

Outbreaks (O)	O1	O2	O3	O4	O5	O6	O7	O8	O9	O10	O11	O12	Total
Lung	2	2	3			2	2	3	3	3	2	3	25
Kidney		2			3	2				3	2		12
Spleen				4									4
Oro-cloacal swab				2								3	5
Positive (RT-qPCR)	2	2 (1 lung +1 Kidney)	3	6	2	3 (2 lungs +1 Kidney)	2	3	3	4 (2 lungs +2 Kidneys)	4	5 (2 lungs + 3 Oro-cloacal swabs)	39/46 (84.8%)

O, outbreak.

### 4.5 Amplification of S1 gene by single-step RT-PCR

**Figure 4:**
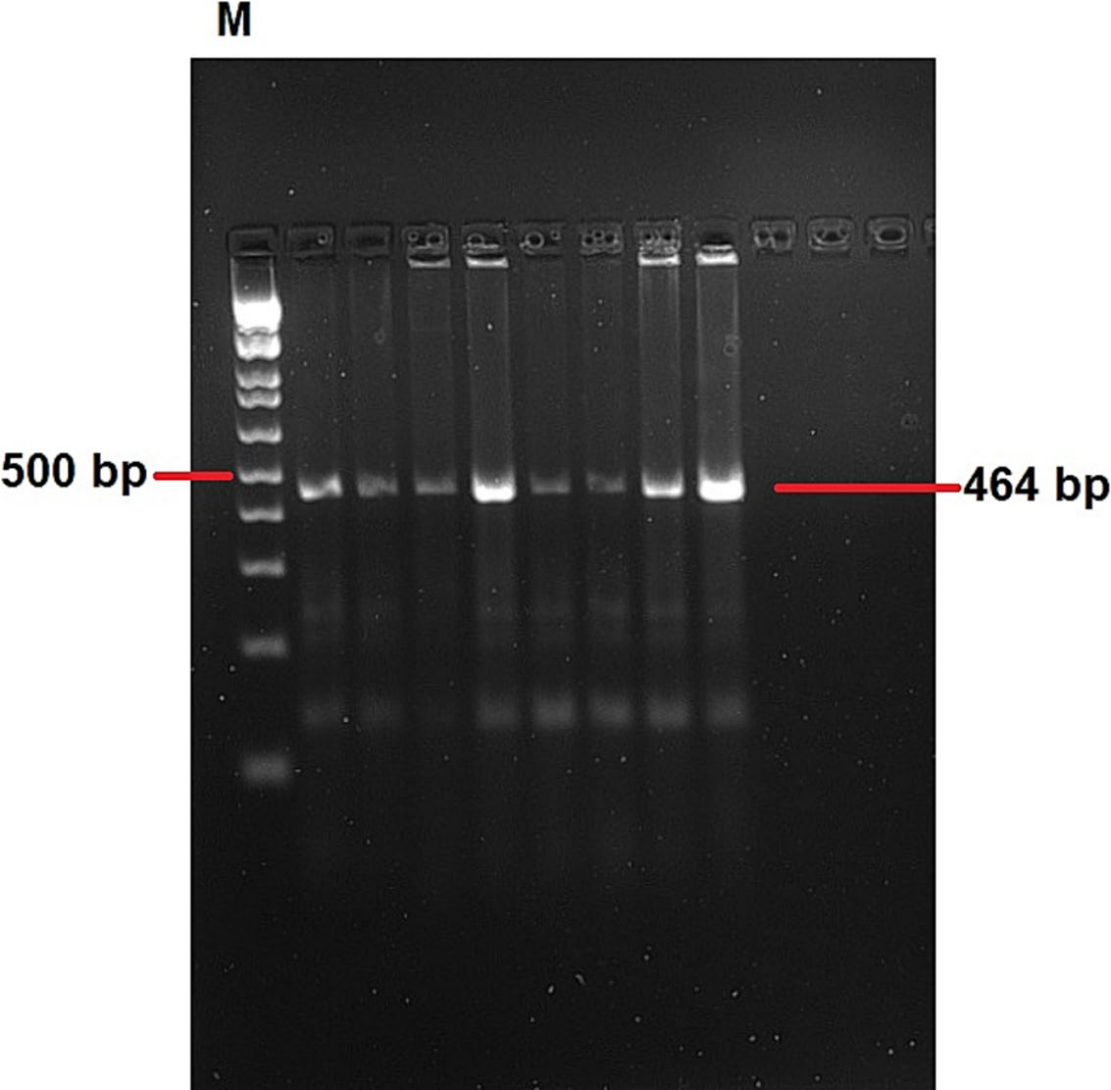
A representative agarose gel electrophoresis showing RT-PCR products of approximately 464 bp of the targeted region. Lane M: 100 bp ladder. The remaining gel images were provided as a Supplementary material 1.

In total, 22 out of the 39 IBV RNA positive samples (56.41%) were selected for single-step RT-PCR based on the Ct value. These samples comprised 12 from lung, 4 from spleen, 3 from kidney, and 3 from oro-cloacal swab, representing 11 outbreaks. The field samples yielded the expected ~464 bp amplicon of the S1 gene. Representative amplicons with expected band size of 464 bp are shown in (Figure 4), while remaining gel images were provided as a Supplementary material 2.

### 4.6 Sequencing of S1 gene PCR products, BLAST and pairwise analysis

Twenty-two S1 gene PCR products were sequenced, and the consensus sequences have been deposited in the NCBI nucleotide GenBank database with accession numbers OR161340 - OR161361. According to BLAST analysis, these sequences showed a 96.25 to 100% nucleotide similarity to different reference strains of IBV available in the NCBI nucleotide database.

When comparing UAE-IB viruses between different lineages, substantial sequence diversity was observed, ranging from 77 to 81%. Specifically, viruses in the GI-1 lineage differ by 77% in their nucleotide sequences from those in the GI-13 lineage and by 80% from those in the GI-23 lineage (Table 4). Viruses in the GI-13 lineage differ by 80–81% from those in the GI-23 lineage. UAE-IB viruses within the same lineages showed high similarity, ranging from 99 to 100%. For example, viruses within the GI-1 lineage (OR161341 and OR161342) are 100% identical in their nucleotide sequences, originating from the same outbreak in farm C. Viruses within the GI-23 lineage (OR161340 and OR161347) are 99% identical, having been sequenced from different outbreaks in August and December 2022 in the same farm (farm A) (Table 2). Sequences within the GI-13 lineage are 100% identical in their nucleotide sequences, despite being reported from different outbreaks in various farms (A, C, D, E, F, and G) at different times (Table 4).

**Table 4:** Between and within lineages diversity of UAE-IBV sequences.

	G1-1	GI-13	GI-23
G1-1	100%		
GI-13	77%	100%	
GI-23	80%	80–81%	99%

### 4.7 Phylogenetic analysis of S1 gene sequences

**Figure 5:**
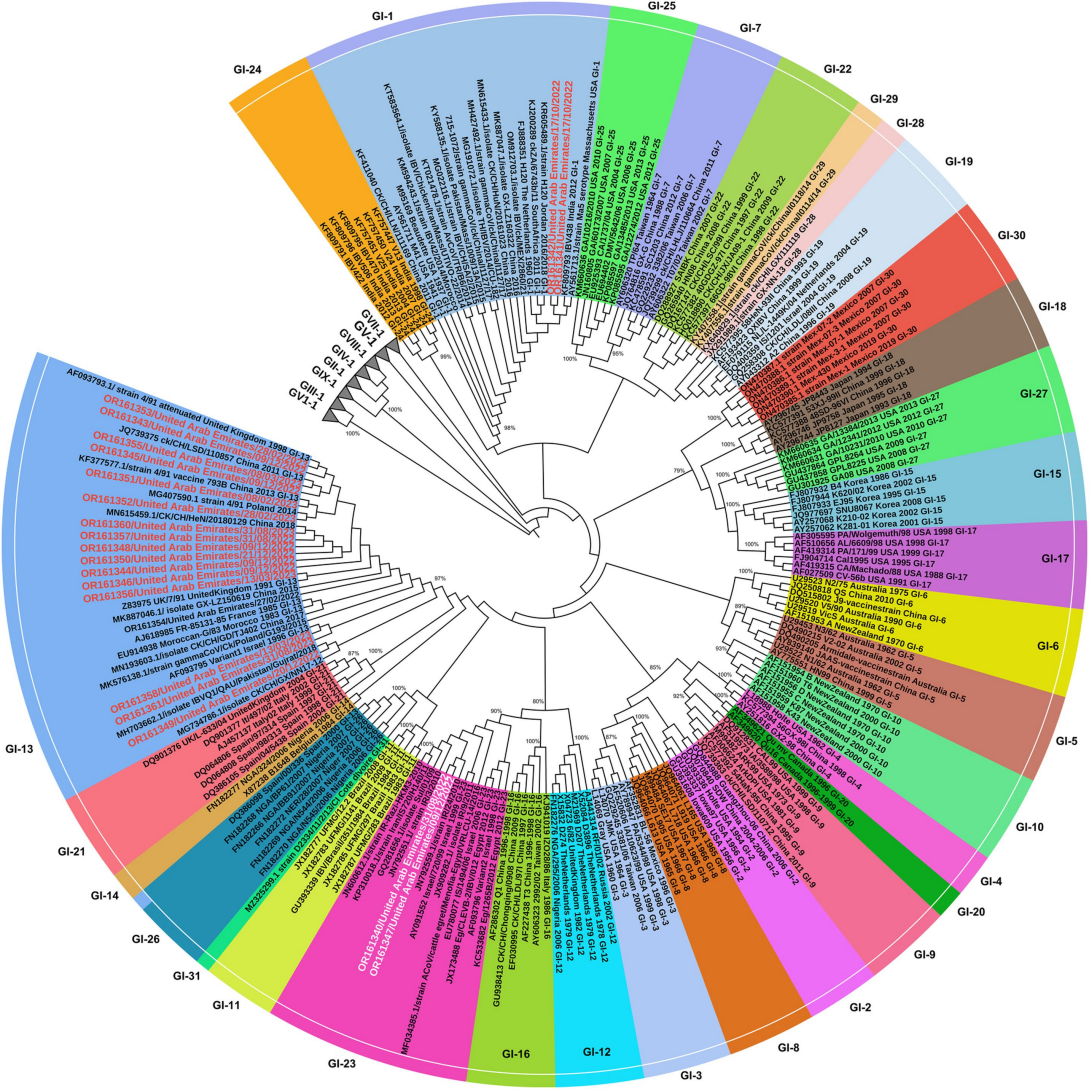
Phylogenetic tree based on alignment of partial S1 gene sequences of 22 UAE IB viruses (in red) and 212 corresponding reference sequences of isolates/strains (vaccines or field strains) retrieved from GenBank (in black), in total 234 sequences. The phylogenetic tree was constructed with MEGA X software with maximum likelihood of 1,000 bootstrap replicates. GenBank accession numbers are shown along with the strains. All 9 groups of IBV were presented. The IBV group-1 including the 31 lineages are indicated [GI (1-31) lineages], while other lineages (GII-GIX) are collapsed. UAE-IB viruses were written in red text in GI-13 & GI-1 and in white text in GI23.

Genotyping of 22 IB viruses detected in this study, was performed by phylogenetic analysis of the S1 gene sequences of 208 strains representing all currently available IBV lineages worldwide. The analysis revealed that 18 out of 22 (81.8%) sequences of IBV S1 gene obtained in this study clustered within the GI-13 lineage that include strains circulating in Poland, the United Kingdom, China, Pakistan, Israel, France, Morocco, and Spain. The UAE-IB viruses within this lineage represent the dominant genotype associated with IB infections (Figure 5). Moreover, two out of the 22 (9.1%) sequences (OR161341 and OR611342) were clustered within GI-1 lineage, while the remaining two out of 22 (9.1%) sequences (OR161340 and OR611347) were classified within GI-23 lineage (or Middle East IBV serotype) harboring strains circulated in Iran, Egypt, and Israel.

### 4.8 Differentiation of filed versus vaccine strains of IBV

The nucleotide and amino acid identity of the UAE IB viruses within each lineage were evaluated against the corresponding vaccine strain in the same lineage, or both vaccine strains, in attempts to classify the UAE IB viruses into field and vaccine strains following the criterion mentioned in the methodology.

The UAE viruses OR161340 and OR161347, classified within GI-23 (Middle East IBV serotype), were compared to both vaccine strains used in the UAE (4/91 and Massachusetts serotypes). However, only strain 4/91 vaccine (793B) from China (Acc. KF377577.1) and Ma5 strain serotype from Massachusetts USA (Acc. AY561713) was shown in Table 5. More field strains versus vaccine strains comparison were shown in Supplementary material 1. The UAE viruses shared 80–81% identity at nucleotide and amino acid levels when compared to the vaccine strain 4/91. Similarly, these strains showed 80% nucleotide identity and 76–77% amino acid identity when compared to the Ma5 strain. In both cases, the homology is less than 99%, hence they were considered as field strains.

**Table 5:** Nucleotide (304 bp) and amino acids (108 aa) identity between UAE-IB viruses and the corresponding common vaccines used in UAE (4/91) live-attenuated vaccines which represented by Strain 4/91 vaccine (793B) from China (Acc. KF377577.1) and Mass-type live-attenuated which represented by strain Massachusetts USA (Acc. AY561713) was shown.

UAE viruses identified in this study (total = 22 strains)	Lineage belongs to	Strain 4/91 vaccine (793B) from China (Acc. KF377577.1)		Strain Ma5 serotype Massachusetts USA (Acc. AY561713)		Classification
		nt identity (324 nt)	aa identity (108 aa)	nt identity (324 nt)	aa identity (108 aa)	
OR161340 OR161347 (*n* = 2)	GI-23 (Middle East serotype)	80–81%	80–81%	80%	76–77%	Field strain
OR161341 OR161342 (*n* = 2)	GI-1 (Massachusetts serotype)	77%	72%	100%	99%	Vaccine strain
Remaining UAE viruses (18 strains) (*n* = 18)	GI-13 (4/91 serotype)	100%	100%	78%	73%	Vaccine strain

nt, nucleotide; aa, amino acid.

The UAE viruses (OR161341 and OR161342) classified within GI-1 (Massachusetts IBV serotype) showed complete nucleotide identity (100%) to the Ma5 vaccine strain and share 99% identity at the amino acid level. Therefore, they were considered as vaccine strains.

The remaining UAE viruses identified in this study (18 strains sharing 99–100% identity within the sequences) classified within GI-13 (4/91 IBV serotype) were 100% identical to 4/91 vaccine strain at both nucleotide and amino acid levels. Therefore, they were considered vaccine strains.

## 5 Discussion

Widespread vaccinations practices against IB based on the Massachusetts and 4/91 serotypes are adopted by farmers in UAE. Despite this, cases of IB were frequently reported, resulting in substantial economic losses between August 2022 to May 2023. Therefore, this study intended to characterize the virus to support control measures in the country.

In this study, the pathological changes observed in necropsied chickens infected by IB were mostly in the respiratory system and kidneys. Evidence of respiratory lesions particularly fibrinous diphtheric plugs in the trachea, lung congestion, and renal urate deposits are consistent with the findings previously described (13, 14). The histopathological lesions in the kidneys, spleen, trachea, and lung sections were also similar to the previously reported findings (13, 15). Additionally, the PCR analysis revealed the presence of IBV in 84.6% (39/46) of the tested samples, which is a higher rate compared to previous reports from Saudi Arabia (36.5–42.7%) (40, 48) or in South Iraq (74%) (43), Egypt (64%) (49), Jordan (60%) (33), western Europe (59%) (9), Iran (52%) (50), eastern Iran (37.5%) (51), Russia (34%) (5), and middle-south Iraq (32%) (52). It’s important to interpret these results with caution since the samples were collected only from broilers showing clinical signs of respiratory infection and some of the strains are vaccine related.

In the control of IBV, the most common approach involves regular vaccination and the implementation of appropriate biosecurity measures (4). However, this approach is hampered by the high genetic variability of the virus, due to both mutation and recombination events, leading to the constant emergence of new variants with generally poor cross-protection (53). To understand the significance of the variation in the S1 sequences between field and vaccine strains of IBV, we first genotyped the UAE-IBV. The phylogenetic analysis revealed that the UAE IB viruses fell into three lineages within genotype I: GI-1 (vaccine strain, *n* = 2), G1-13 (vaccine strain *n* = 18) and GI-23 (field strain, *n* = 2). Specifically, two UAE-IB viruses belonged to GI-1, 2 strains to GI-23, and the remaining 18 viruses belonged to GI-13. In total, 18/22 (81.8%) UAE-IB viruses are classified within vaccine strains lineages (GI-1) and (GI-13) and likely to be vaccine virus. This was evidenced by their sharing of 99–100% identity with the vaccine strains in the S1 spike gene sequence analyzed (Table 5). This situation may be attributed to the frequency of live IBV vaccination schedules, variations in management practices, and, most importantly, the immune status of the flocks. However, the high vaccine strains detected in this study are not surprising as the live IBV vaccines have been reported to persist in chicks for many weeks after administration (54, 55) or revert to cause outbreaks (19, 20). Indeed, in some studies vaccines-related strains accounted for approximately half or more of the total detections (41, 56). According to our outbreak history, it was evident that the chickens were vaccinated without the introduction of unvaccinated flocks into the farm. This further supports the possibility of these strains being the same vaccine strain.

The study observed significant clinical signs, but it is challenging to determine through molecular analysis whether the detected strains are truly pathogenic or a vaccine-derived virus found along with other disease-causing agents (57). Furthermore, there are currently no known genetic markers consistently capable of distinguishing between vaccine and field strains (57). While the present study employed a combination of phylogenetic and epidemiological criteria to confidently classify sequenced strains, the possibility of misclassification remains due to the circulation of actual field strains closely related (at least in the considered genomic region) to vaccine viruses, warranting further comprehensive evaluations, possibly based on the entire S1 gene sequence (58). The later assumption was also compromised by Callison et al., who showed that in a case of 4/91, the nucleotide sequences of the entire S1 spike gene of the vaccine strain and the pathogenic virus differed by only 0.6% (9), or even there was no difference at all over the section of the gene that was sequenced by Worthington and his colleagues (9).

The persistence of vaccine strains presents several challenges, as it makes diagnosing IBV more complicated. This is because many detected strains often closely resemble or match the homologous vaccines used (57). Furthermore, it hampers the interpretation of the epidemiological scenario, thereby hindering the planning of effective control strategies within the region.

The two IBV strains detected in this study (OR161340 and OR161347) belonged to the GI-23 lineage which represents a cluster of unique wild-type viruses geographically limited to the Middle East (24). The S1 sequences from these viruses showed maximum amino acid identities (80–81%) with the 4/91 serotype and (76–77%) to Ma5 strains, hence considered field strains (wild type or non-vaccine viruses). Despite the vaccination programs adopted by farmers in the Abu Dhabi Emirate, there have been continuous reports of IB-suspected cases. The low correlation in sequence identity between the two IB viruses detected in this study, and the vaccine strains may partially explain the failure of the vaccination programs to control IBV in these flocks due to the low cross-immunity between the field and vaccine strain (59, 60), resulting in high IB-induced morbidity and mortality within vaccinated chicken flocks.

The strains identified in this study across various lineages show a wide range of genetic diversity, indicating multiple evolutionary origins. However, strains within the same lineage are identical, even when observed in different outbreaks or times within or between farms suggesting a common source and/or inter-farm transmission of a single strain. This is likely facilitated by the proximity of some Farms. For example, distances between Farms E and A is 1.2 KM, E and F are 4.8 KM, and G and B is 8.3 KM (Figure 1). Contaminated feces, feed, and drinking water can serve as sources of IBV infection or reinfection during the recovery phase, as the virus can persist in feces for a considerable period. Additionally, indirect transmission can occur through contaminated litter, footwear, clothing, utensils, equipment, and personnel, all of which have been implicated in the spread of IBV over long distances (61, 62). Therefore, strict application of biosecurity measures in poultry farms is crucial. A complex scenario was observed when two lineages (GI-13 and GI-23) were detected in the same farm (farm A), or repeated infections in the same farm such as farm A as well, further highlighting the necessity for enhanced biosecurity measures. Although the partial gene (464 bp) was used to genotype the IBV, and provided useful information, the whole S1 gene (~ 1,620 bp) is recommended to be used for IBV genotyping studies to cover the HVR3 (positioned between 820 and 1,161 of the S1 gene) while characterizing the virus (24).

The present study demonstrated a complex epidemiological situation, with both field (9.1%) and vaccine strains (90.9%) coexisting in the region. The latter being the vast majority, which raises concerns about the pros and cons of a widespread or inadequately planned vaccine application besides the direct cost of vaccine-induced clinical signs.

## 6 Conclusion

This is the first comprehensive study to describe and genotype IBV in the UAE. It holds significant practical implications by providing up-to-date information on the lineages currently circulating in the Abu Dhabi Emirate, albeit limited to clinical data. This study can aid in the selection of the best vaccine for use in the Abu Dhabi region, and in mitigating the impact of uncontrolled circulation of vaccine-derived strains on IBV diagnostics and evolution.

In this study, we have identified three IBV lineages currently circulating in broiler farms across the Abu Dhabi Emirate. These lineages consist of two vaccine lineages: GI-13 (81.8%) and GI-1 (9.1%) in addition to one field lineage GI-23 (9.1%). It is worth noting that vaccine strains (90.9%) predominate over field strains (9.1%). Field genotypes, which do not have corresponding vaccines available, are expected to continue causing IBV outbreaks resulting in economic losses. Identifying IBV field genotypes is of economic importance for making the best use of existing live vaccines. However, continuous surveillance and characterization of new IBV strains is crucial not only in Abu Dhabi Emirate but throughout the UAE for monitoring strain prevalence and the emergence of potentially significant viruses. Such surveillance facilitates understanding the molecular evolution of different genotypes and is crucial for selecting candidate virus strains for the development of new vaccines that match the local circulating field strains.

## Data Availability

The partial S1 gene of IBV UAE strains sequences generated in this study are available in the NCBI nucleotide database under accession numbers from OR161340 to OR161361.

## Footnotes

^1^ http://www.ncbi.nlm.nih.gov/blast
