# Supplementary figures and images for: Clinical, pathological, and genotypic analysis of infectious bronchitis virus in broiler chickens in the Abu Dhabi Emirate, United Arab Emirates
Source: Front Vet Sci. 2025 Jan 27;11:1474181. doi: 10.3389/fvets.2024.1474181 (PMC11808912; doi:10.3389/fvets.2024.1474181)

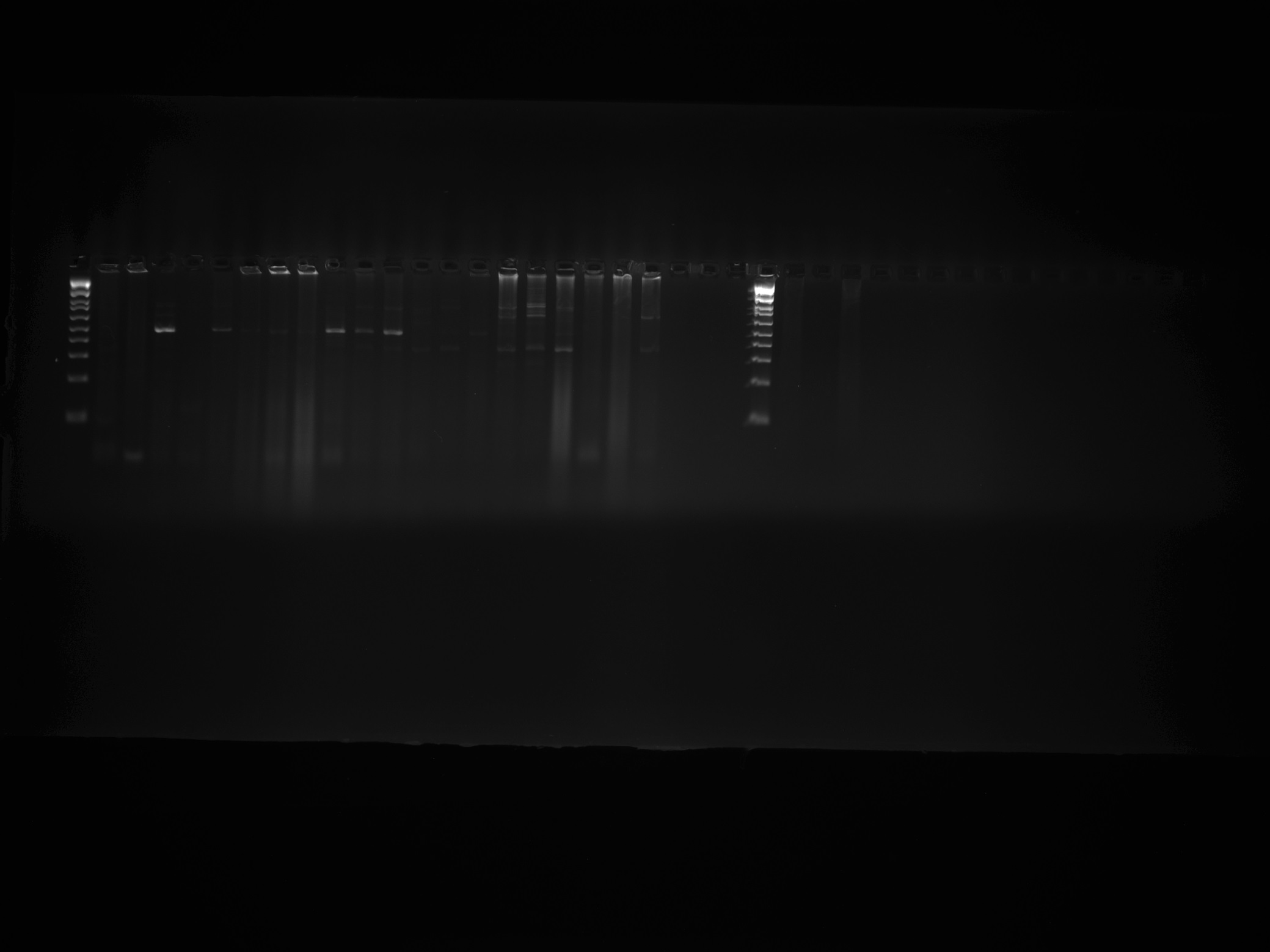

Supplement: Supplementary file 2 [file Data_Sheet_1.zip › Supplimentary-2/IBV_GEL-1.jpg]

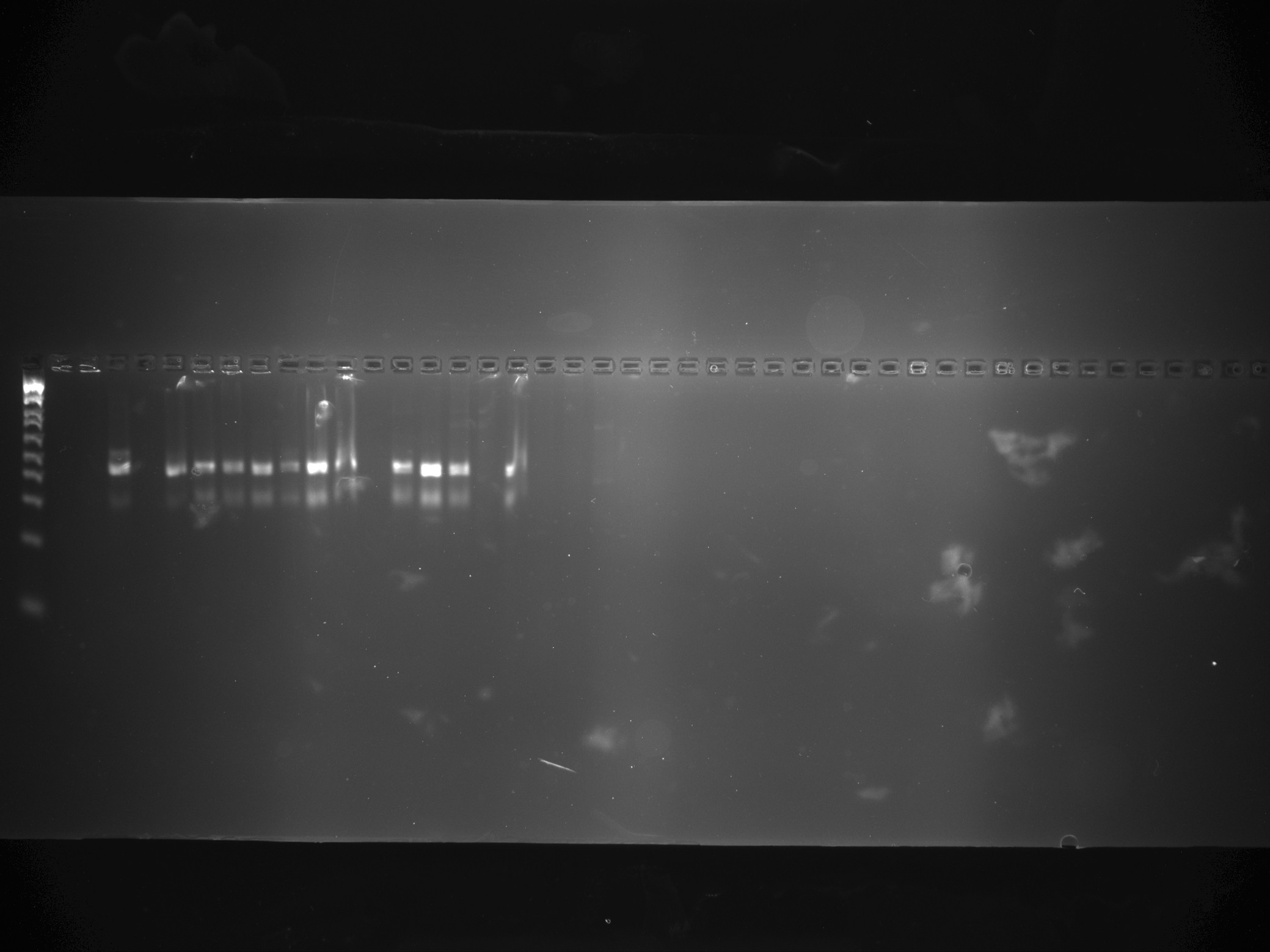

Supplement: Supplementary file 2 [file Data_Sheet_1.zip › Supplimentary-2/IBV_GEL-2.jpg]

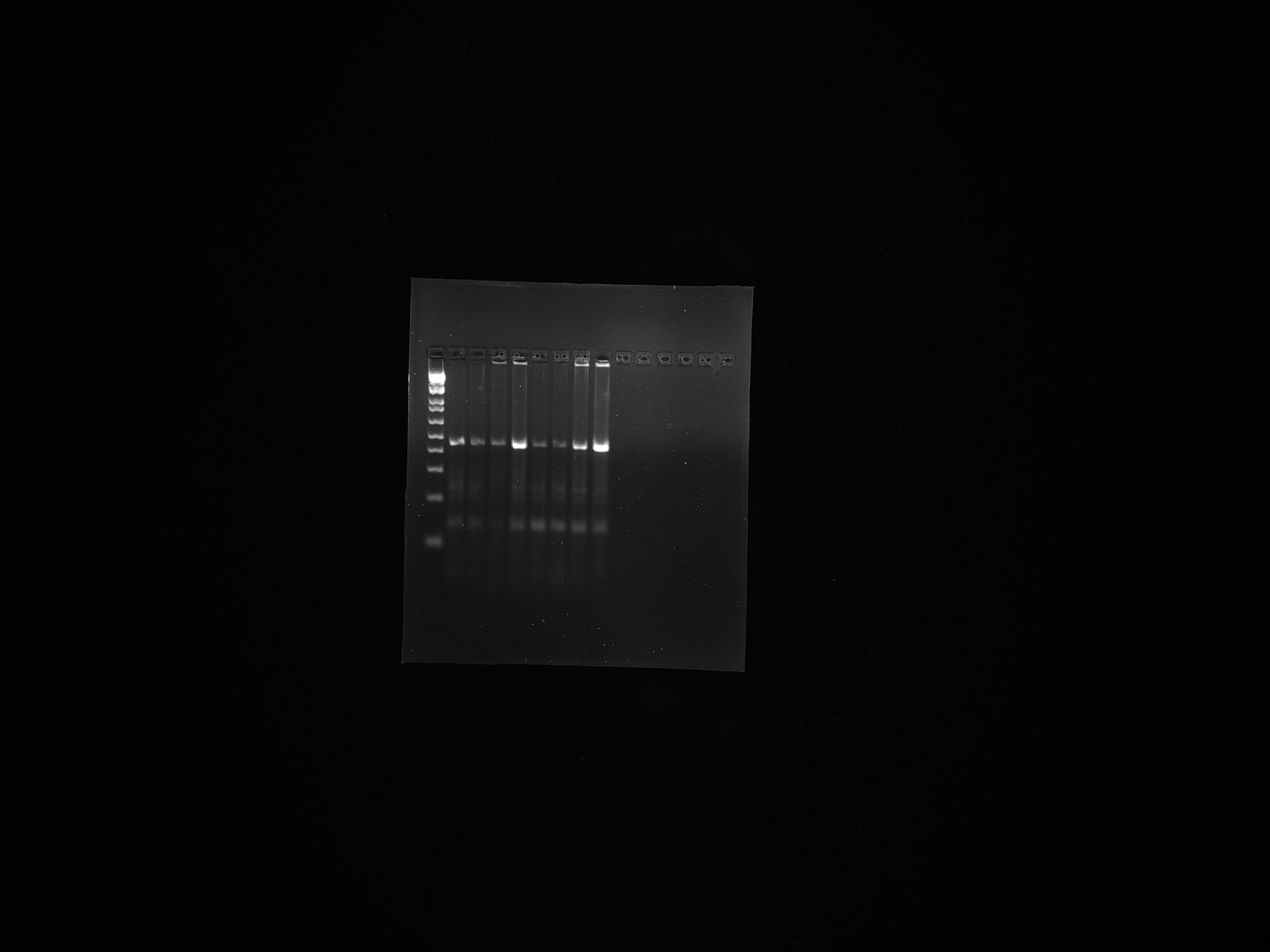

Supplement: Supplementary file 2 [file Data_Sheet_1.zip › Supplimentary-2/IBV_GEL-3.jpg]

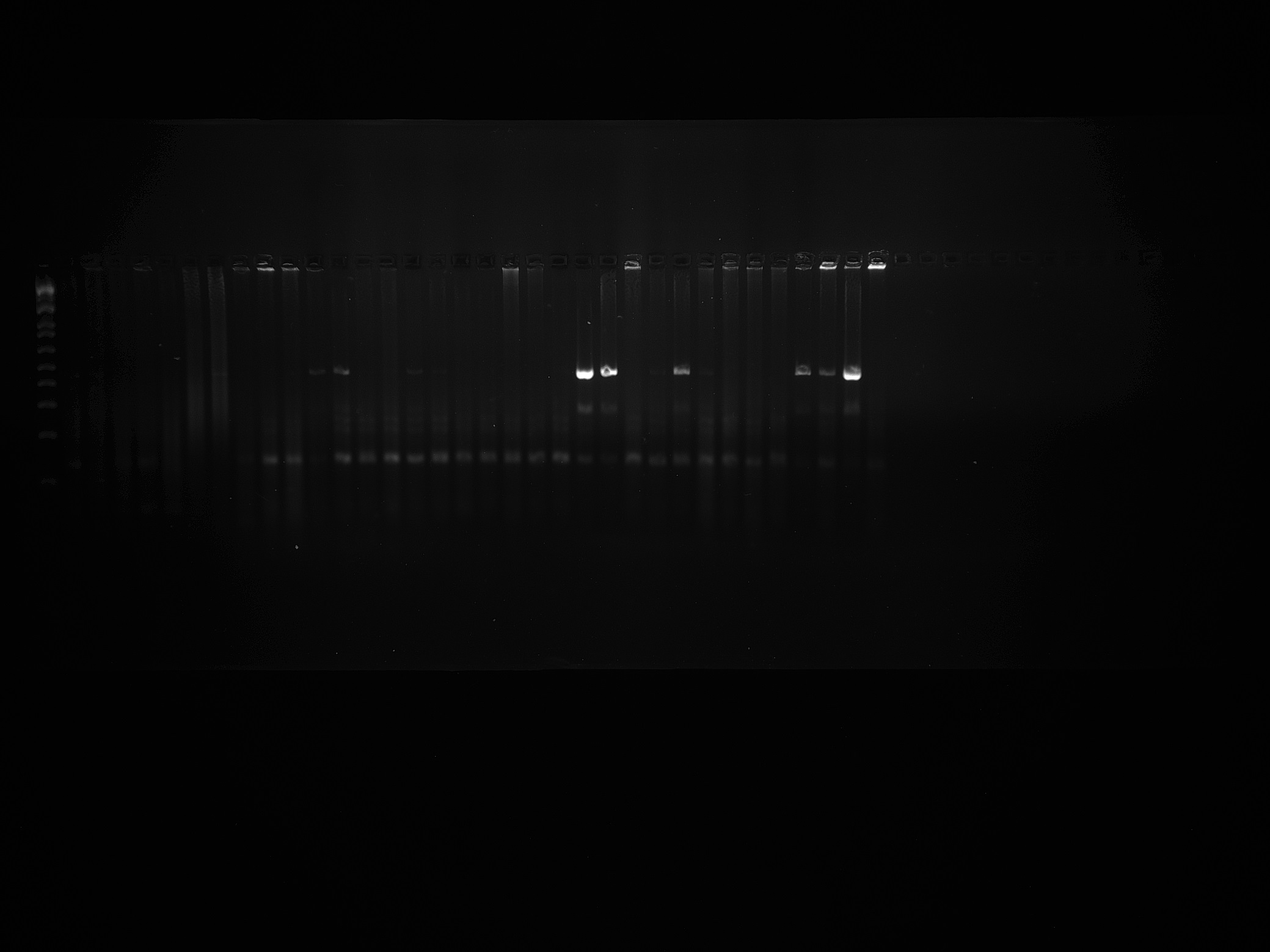

Supplement: Supplementary file 2 [file Data_Sheet_1.zip › Supplimentary-2/IBV_GEL-4.jpg]
